# Engineering Magnetic Beads for Affinity Enrichment of Exosomes

**DOI:** 10.34133/csbj.0170

**Published:** 2026-07-22

**Authors:** Xu Wang, Baiqing Li, Di Wu, Xiaotong Cen, Dajiang Qin

**Affiliations:** ^1^ Key Laboratory of Biological Targeting Diagnosis, Therapy and Rehabilitation of Guangdong Higher Education Institutes, The Fifth Affiliated Hospital, Guangzhou Medical University, Guangzhou, China.; ^2^ Guangzhou National Laboratory, Guangzhou, China.; ^3^ Centre for Regenerative Medicine and Health, Hong Kong Institute of Science & Innovation, Chinese Academy of Sciences, Hong Kong SAR, China.

## Abstract

Exosomes play a crucial role in intercellular communication and disease diagnostics, yet their affinity enrichment remains challenging due to the heterogeneous expression of surface markers. To address this, we engineered a recombinant fusion protein, ExoBp, designed to bind multiple exosome surface biomarkers through integrated peptide motifs and protein domains with sterically unhindered conformation revealed by structure prediction. The constructed protein was expressed but required denaturing conditions for purification. Despite refolding challenges, ExoBp was successfully immobilized onto streptavidin-coated magnetic beads in its denatured state, followed by on-bead refolding to restore functionality. The resulting ExoBp magnetic beads demonstrated efficient exosome binding, as confirmed by the detection of exosomal markers after coincubation with exosome derived from different types of cells. Both dialysis- and dilution-based refolding methods yielded stable ExoBp-bead complexes with retained binding capacity. This study presents a novel, affinity-based exosome enrichment tool leveraging multitarget binding, offering a promising platform for exosome isolation and downstream biomedical applications.

## Highlights

• Bioengineering protein with multiple recognition sites of exosome (ExoBp) was designed
• ExoBp was immobilized on magnetic beads by affinity to generate exosome affinity magnetic beads
• Exosomes can be enriched on ExoBp magnetic beads

## Graphical Abstract

**Figure:**
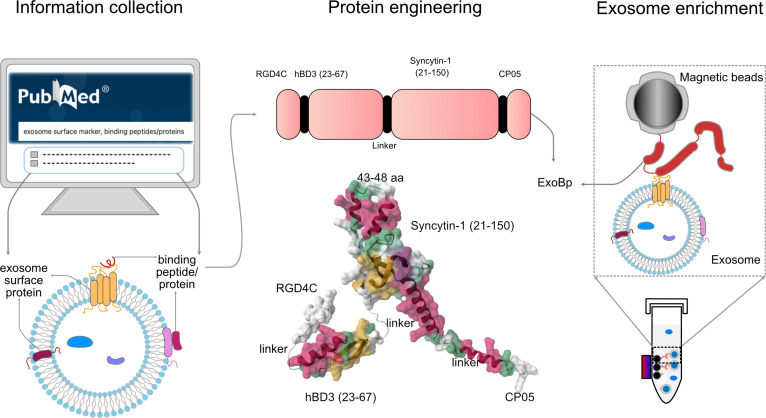

## Introduction

Extracellular vesicles (EVs) are cell-secreted, nano-scaled lipid-bound vesicles that mediate intercellular communication by transporting bioactive molecules across biological barriers, such as the blood–brain barrier, thereby supporting neuroprotection, angiogenesis, and other physiological processes [1,2]. EVs are broadly classified into exosomes, microvesicles, and apoptotic bodies based on their size, biogenesis, molecular markers, and functional pathways [3]. According to the 2023 guidelines of the International Society for Extracellular Vesicles (ISEV), vesicles under 200 nm (typically 30 to 200 nm) are defined as exosomes [4]. Exosomes have garnered marked attention due to their well-characterized composition, diagnostic potential, and ease of isolation from accessible biological fluids [5,6]. Their natural biodistribution, tropism, and low immunogenicity further position them as superior drug delivery vehicles compared to synthetic nanoparticles [7]. Current research extensively explores their applications in drug delivery and diagnostics [8,9].

Exosomes possess a proteolipid architecture that encapsulates diverse cargo, including proteins, lipids, DNA, mRNA, microRNA, and noncoding RNAs. Their biogenesis involves a double invagination process: initial plasma membrane invagination forms early endosomes, followed by inward budding of endosomal membranes to generate intraluminal vesicles (ILVs) within multivesicular bodies (MVBs). Upon fusion of MVBs with the plasma membrane, ILVs are secreted as exosomes [10]. The endosomal sorting complex required for transport (ESCRT) machinery (comprising ESCRT-0, -I, -II, and -III)-dependent and independent mechanisms is involved in exosome biogenesis [11–13]. Indirect influences from lysosomal, autophagic, and Golgi-derived vesicle trafficking, as well as cellular states (e.g., stress and genomic integrity), also modulate exosome biogenesis [14]. This intricate network underscores the complexity, diversity and heterogeneity of exosome formation pathways.

Exosomes exhibit inherent heterogeneity in size, content, and function depending on their cellular origin and culture conditions. Tetraspanins (CD9, CD63, and CD81) are widely used as surface biomarkers for exosome purification and characterization, alongside luminal markers such as ALIX, TSG101, flotillins, and heat shock proteins [15–18]. However, these markers lack universal specificity across exosomes from diverse cell types. Recent proteomic analyses identified 22 core proteins, including syntenin-1, as universally enriched in exosomes regardless of origin or isolation method, offering potential pan-exosome biomarkers [19].

Exosome isolation strategies rely on physical properties or affinity-based approaches. Though several approaches including commercial exosome isolation kits are used, ultracentrifugation (UC) remains the gold standard, pelleting exosomes after preclearing cellular debris [20,21]. Density gradient centrifugation enhances purity but struggles to resolve EVs with overlapping densities [22]. Size-dependent methods, such as size exclusion chromatography, ultrafiltration, and asymmetrical flow field-flow fractionation, exploit vesicle size or Brownian motion [23,24]. Affinity-based techniques employ antibodies, aptamers, or peptides targeting surface markers (e.g., CD9, CD63, and EpCAM) but are limited by cost and specificity [25–27]. Microfluidic devices integrate multiple separation principles but require specialized equipment [28]. No single method achieves ideal yield and purity; hybrid approaches are recommended to balance practicality and performance.

**Fig. 1.**
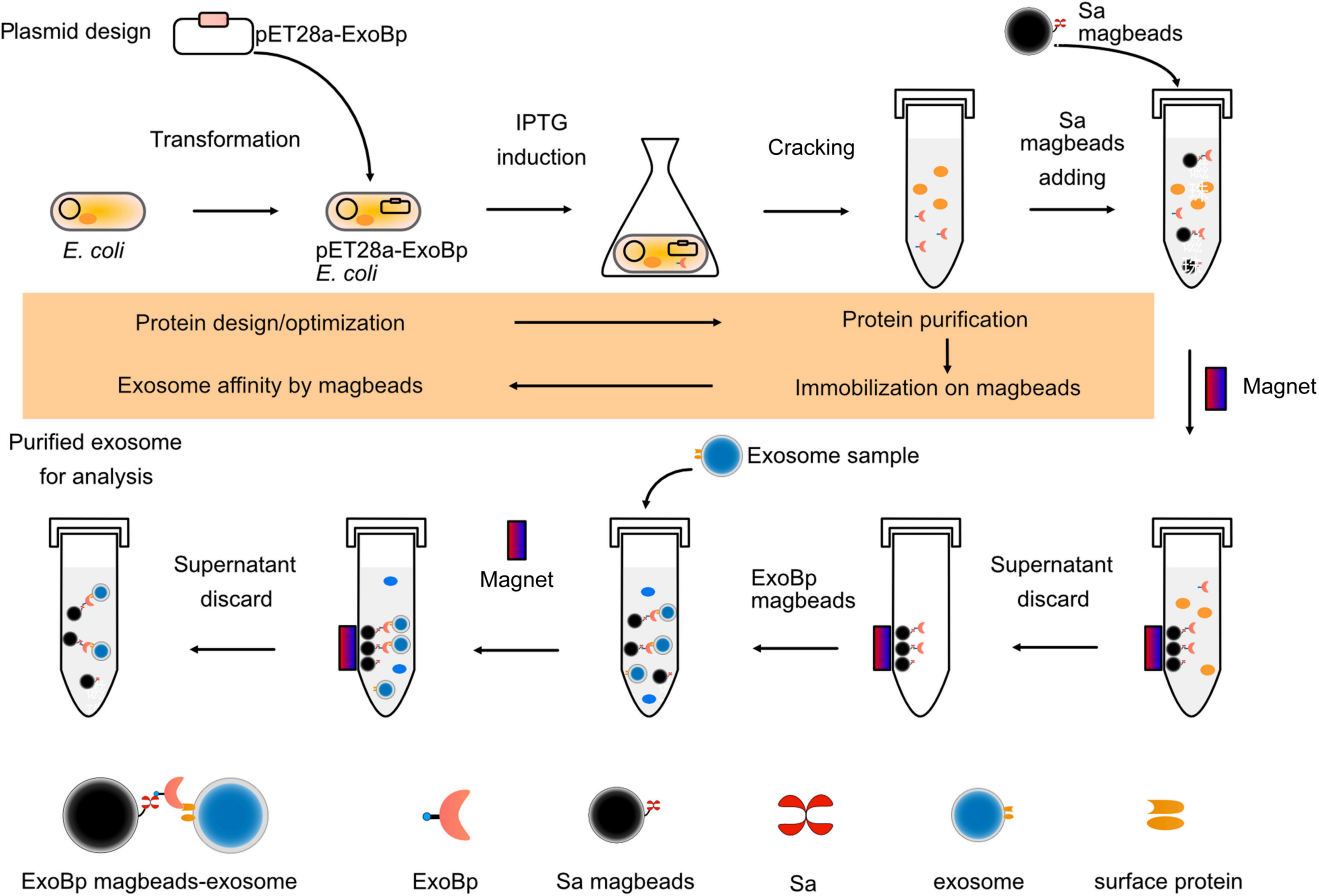
Schematic diagram for platform of exosome enrichment by engineered magnetic beads. Recombinant exosome-binding protein (ExoBp) with multiple recognition sites of exosome was designed and expressed in *E. coli* with plasmid by isopropyl-β-D-thiogalactopyranoside (IPTG) induction. The cell lysate was incubated with streptavidin-conjugated magnetic beads (Sa magbeads) to generate ExoBp-linked Sa magbeads (ExoBp magbeads) and then the magbeads were harvested with a magnet. After incubating the ExoBp magbeads with the exosome-containing fluid, exosome was enriched on magnetic beads. The purified exosome was used for further analysis.

In this study, we present a multiple-site recognition, magnetic bead-based strategy for exosome enrichment (Fig. 1). A recombinant protein engineered with 4 exosome surface marker recognition sites was immobilized on magnetic nanoparticles. This system demonstrates efficient exosome enrichment, offering a promising tool for exosome isolation and analytical applications.

## Materials and Methods

### Plasmid and bacteria

The amino acid sequences of human Beta-defensin 103 (hBD3) and Syncytin-1 were retrieved from the UniProt database (UniProt number P81534 for hBD3 and Q9UQF0 for Syncytin-1). Sequences for the RGD4C and CP05 peptides, along with linker regions, were derived from published references [29, 30] (Table S1). The full ExoBp protein sequence was designed by concatenating these components, and the corresponding DNA sequence was reverse-translated and codon-optimized for *Escherichia coli* expression (Table S2). The synthesized DNA was cloned into the pET-His-Strept Tag II plasmid using Gibson assembly (seamless cloning). The recombinant plasmid (pET-His-Strept Tag II-ExoBp) was transformed into *E. coli* BL21(DE3) cells for protein expression.

### Expression and purification of ExoBp

A single colony of *E. coli* BL21(DE3) harboring the ExoBp plasmid was inoculated into 5 ml of LB medium and incubated at 37 °C with shaking (200 rpm) until reaching an OD_600_ (optical density at 600 nm) of 0.6 to 0.8. Protein expression was induced with 0.5 mM isopropyl-β-D-thiogalactopyranoside (IPTG), followed by overnight incubation at 16 °C. Cells were pelleted (6,000 × *g*, 10 min, 4 °C), resuspended in binding buffer (50 mM NaH_2_PO_4_, 300 mM NaCl, and 40 mM imidazole, pH 8.0), and lysed via high-pressure homogenization. Lysates were centrifuged (8,000 × *g*, 20 min, 4 °C), and the clarified supernatant was filtered (0.22 μm) and loaded onto a pre-equilibrated Ni-NTA (TIANGEN, #PA201-01). After washing with binding buffer, bound proteins were eluted using elution buffer (50 mM NaH_2_PO_4_, 300 mM NaCl, and 250 mM imidazole, pH 8.0). For denaturing purification, all buffers contained 8 M urea. Refolding was attempted via stepwise dialysis against phosphate-buffered saline (PBS) with decreasing urea concentrations (4, 2, 1, 0.5, and 0 M).

### Immobilization of ExoBp on streptavidin-modified magnetic beads

Streptavidin-coated magnetic beads (Sa magbeads, MedChemExpress, #HY-K0208) were equilibrated in urea-containing binding buffer (8 M urea). Purified ExoBp (1 ml) was incubated with 20 μl of beads (200 μg) for 1.5 to 2 h at room temperature. Beads were magnetically separated, washed 5 times with binding buffer, and resuspended in 20 μl of PBS. Immobilization efficiency was assessed via sodium dodecyl sulfate–polyacrylamide gel electrophoresis (SDS-PAGE). Control experiments used bovine serum albumin (BSA) instead of ExoBp.

### Refolding of immobilized ExoBp

For dilution-based refolding, ExoBp-bound beads in 8 M urea were dropwise diluted into 20 ml of PBS and gently agitated for 1 h at room temperature. For dialysis-based refolding, beads were suspended in 1 ml of 8 M urea, loaded into a dialysis bag, and dialyzed against PBS with a stepwise urea gradient (4, 2, 1, 0.5, and 0 M; 2 h per step). Refolded beads were washed and resuspended in PBS.

### Exosome isolation

Human umbilical cord-derived mesenchymal stem cells (hUMSCs) were isolated and cultured according to a previous study [31]. hUMSCs and HEK293T cells (ATCC: CRL-3216) were cultured in complete Dulbecco’s modified eagle medium DMEM (with 10% fetal bovine serum) in 15-cm dishes until 90% to 100% confluent. Cells were washed with PBS and incubated in serum-free DMEM for 24 to 48 h to collect conditioned medium. The 25-ml medium was sequentially centrifuged (200 × *g*, 5 min; 10,000 × *g*, 30 min; 4 °C) to remove cells and debris. Exosomes were pelleted via UC (120,000 × *g*, 70 min, 4 °C), washed in PBS, and resuspended in 250 μl of PBS.

### Transmission electron microscopy

For transmission electron microscopy (TEM) imaging of exosome, isolated exosome was thoroughly mixed to ensure uniform dispersion without visible aggregation. A 300-mesh carbon-coated copper grid was rinsed twice with ultrapure water and air-dried. A 10-μl aliquot of exosome was deposited onto the carbon film side of the grid and allowed to adsorb for 5 min at room temperature. Two microliters of 2% uranyl acetate was applied to the grid surface for negative staining for 2 min, and the excess staining solution was gently removed. The grid was air-dried for 30 min and examined by TEM (Tecnai G2 Spirit).

### Nanoparticle tracking analysis

Isolated exosome was detected using a Malvern Panalytical NanoSight NS300 (Malvern), and data were analyzed using nanoparticle tracking analysis (NTA) software.

### Exosome binding with ExoBp magbeads

Fifty microliters (500 μg) of refolded ExoBp magbeads or Sa magbeads was precleaned with PBS. A 200-μl aliquot of exosome and 250 μl of PBS were added into the tube. The mixture was gently vortexed and incubated for 2 h at room temperature. The beads were harvested, washed, and resuspended with 50 μl of PBS. Then, 20 μl of beads and a 20-μl aliquot of exosome were used for immunoblotting.

### Western blotting and antibodies

Sample (0.1 OD_600_ bacteria lysates or a 20-μl aliquot of exosome) was prepared with SDS loading buffer and heated at 95 °C for 5 min. Sample was loaded and separated by SDS-PAGE and then transferred on polyvinylidene fluoride membrane. The membrane was blocked by 5% (w/v) nonfat milk for 1 h at room temperature. The membrane was immunoblotted by primary antibody for 1 h and secondary HRP antibody for 1 h at room temperature. The signal was developed by ECL chemiluminescence reagent and visualized by image system (Tanon 5200). The following antibodies were used for immunoblotting: anti-His-tag (Proteintech, #10001-0-AP, 1:2,000), anti-CD63 (Proteintech, #25682-1-AP, 1:1,000; Santa Cruz, #sc-5275, 1:500), anti-CD81 (Proteintech, #66866-1-Ig, 1:1,000), anti-Alix (Proteintech, #67715-1-Ig, 1:2,000), anti-TSG101 (Proteintech, #84334-4-RR, 1:2,000), and HSP90-β (Cell Signal Technology, #5087S, 1:1,000).

### Protein structure prediction and molecular docking

The SLC1A5 structure (PDB: 6GCT) and Syncytin-1 predicted structure (AlphaFold entry: Q9UQF0) were used for docking studies. ZDOCK software [32] was used for the prediction of interaction interfaces between Syncytin-1 and SLC1A5 (203 to 232 aa). The ExoBp structure was modeled using AlphaFold3 (https://alphafoldserver.com).

## Results

### Design of ExoBp for exosome binding

To develop a magnetic bead-based platform for exosome isolation, we engineered a recombinant multiligand protein (ExoBp) targeting conserved exosome surface biomarkers. While tetraspanins (CD9, CD63, and CD81) are commonly used for affinity isolation of exosome [33], their heterogeneous expression across exosomes from diverse cell types limits universality [34]. To address this, we intended to integrate additional ligands targeting recently identified pan-exosome markers, including SLC1A5, ITGB1, and SLC3A2 [19]. First, we reviewed documented peptides or proteins that bind to exosome surface markers and membrane components (Table S1).

The ExoBp protein was finally designed as a fusion construct comprising (a) Beta-defensin 103 (hBD3), which binds SLC3A2 [35]; (b) Syncytin-1, interacting with SLC1A5 [36]; (c) the CP05 peptide targeting CD63 [30]; and (d) the RGD4C peptide for ITGB1 binding [29]. hBD3 and Syncytin-1 were constructed as the main frame of protein while 2 peptides, CP05 and RGD4C, were recombined to terminus. These components were linked via spacers GS_4_, (GS)_3_ and EA_3_K, with an N-terminal His-Tag-Strept-Tag II for purification and immobilization.

### Optimization of ExoBp protein

**Fig. 2.**
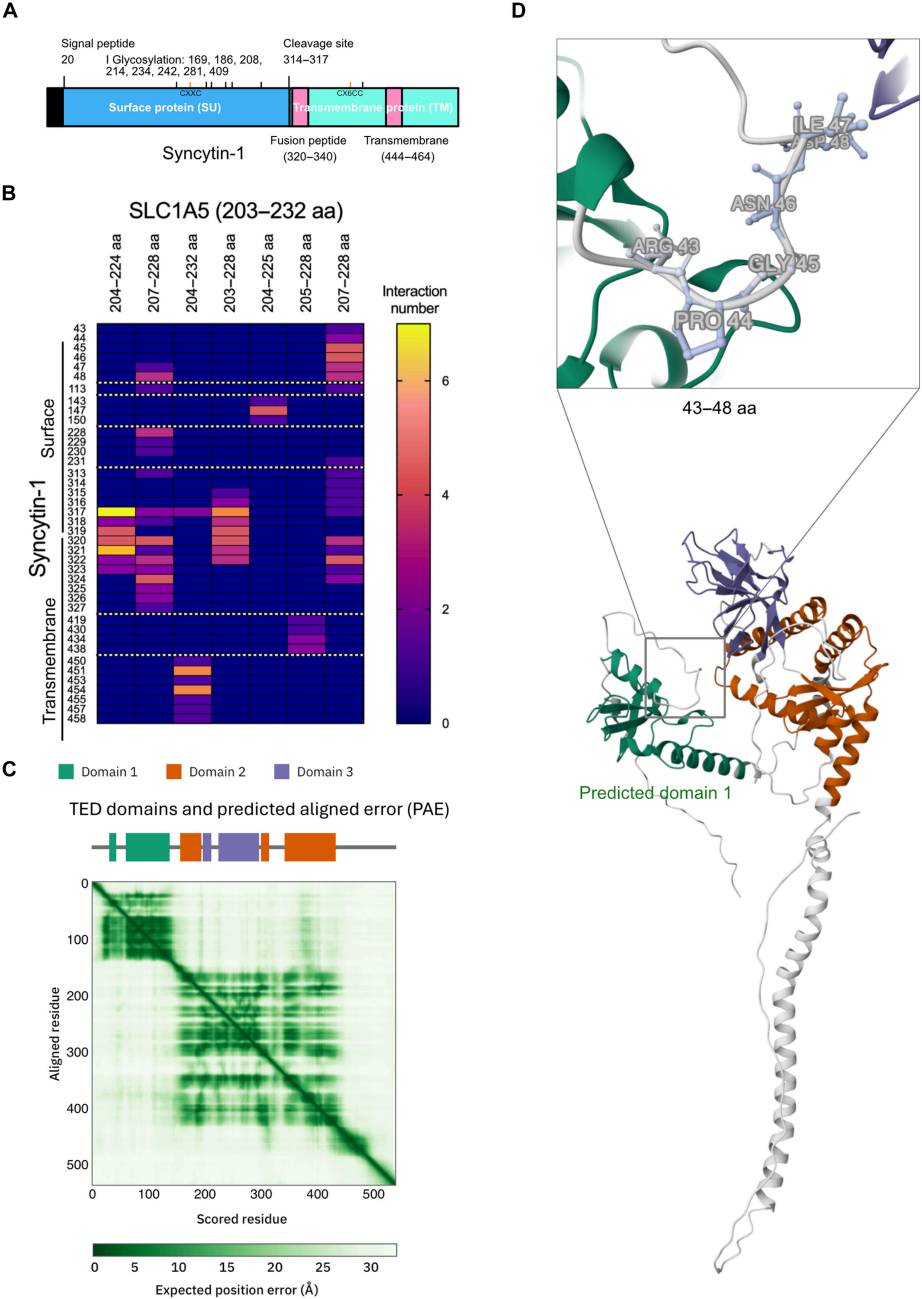
Prediction and analysis of interaction between Syncytin-1 and SLC1A5. (A) Diagram for Syncytin-1 domains including signal peptide, surface protein (SU), fusion peptide, and transmembrane domain (TM). Glycosylation site, cleavage site, and CX6CC site for the interaction of surface protein domain and transmembrane protein domain are presented. (B) Heat map of interaction residue number of Syncytin-1 and SLC1A5 (203 to 232 aa) analyzed by ZDOCK. The 43 to 48 aa region in surface protein domain was implicated to have interaction with SLC1A5 (203 to 232 aa). (C) The encyclopedia of domain (TED) prediction of Syncytin-1. Structure database analysis indicated that Syncytin-1 contains 3 potential domains. (D) Predicted structure of full length of Syncytin-1. Three predicted domains are heighted in colors. Predicted structure of the 43 to 48 aa region in surface protein domain is shown in a magnified structure diagram, reflecting an exposed, flexible region.

**Fig. 3.**
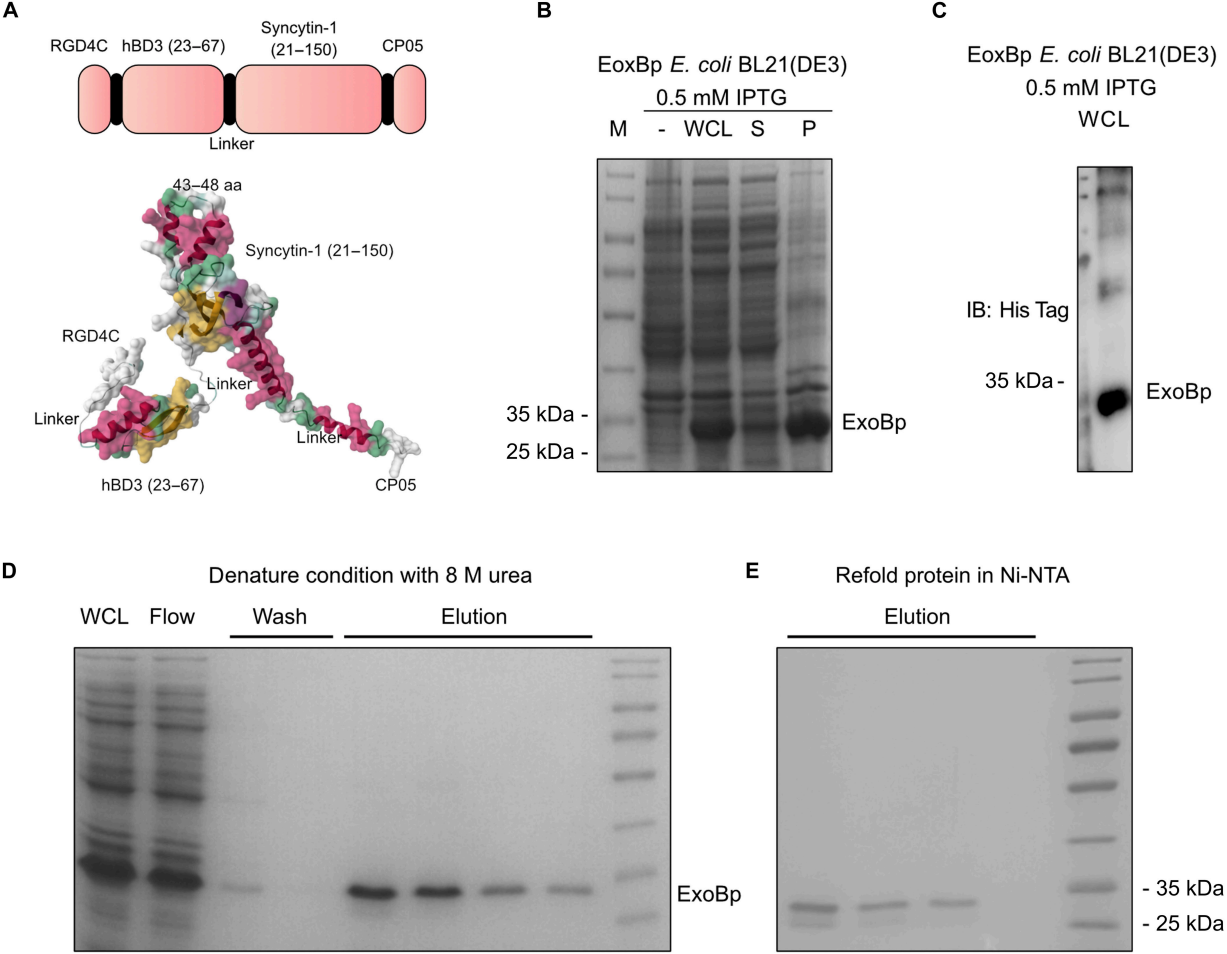
Expression and purification of ExoBp protein. (A) ExoBp contains RGD4C, hBD3 (23 to 67), Syncytin-1 (21 to 150), and CP05, by linkers G_4_S, (GS)_3_, and EA_3_K from N- to C-terminus. The predicted structure of ExoBp represented in the molecular surface model. Four functional regions show exposition instead of self-interaction. The 43 to 48 aa region of Syncytin-1 is not interacting with other regions or embedded in other structures. (B) Induction of ExoBp by IPTG. ExoBp appears to be mostly insoluble. (C) WB detection of ExoBp in cell lysate after induction by IPTG. (D) Purification of ExoBp by Ni-NTA under denature conditions (8 M urea). WCL, whole cell lysate. (E) Purification of ExoBp after refolding protein into PBS buffer on Ni-NTA.

hBD3 is a low-molecular-weight protein with 67 aa, including a signal peptide (1 to 22 aa) [37]. Syncytin-1 is a 538-aa protein comprising a signal peptide, surface domain, cleavage site, fusion peptide, and transmembrane domain [38]. Syncytin-1 interacts with the SLC1A5 203 to 232 region [36]. To design a minimal yet efficient recombinant protein, we analyzed the structure and property of Syncytin-1 (Fig. 2A). We predicted the interaction between Syncytin-1 and the SLC1A5 203 to 232 aa region by ZDOCK (Fig. 2B). The results highlighted 2 flexible regions (43 to 48 aa and 313 to 327 aa) as potential binding interfaces. The former region lies within the surface domain, which is known to be involved in receptor binding, while the latter region spans the cleavage site (314 to 317 aa) and partial fusion region (320 to 340 aa). Moreover, the encyclopedia of domain (TED) prediction revealed that Syncytin-1 has a predicted domain with boundaries 32 to 41 aa and 61 to 137 aa, and the region between these boundaries (43 to 48 aa) is predicted to be responsible for SLC1A5 binding (Fig. 2C and D). In addition, N-terminal 144 amino acids of the mature surface glycoprotein of Syncytine-1 was determined as the minimal receptor-binding domain [39]. The truncated Syncytin-1 (21 to 150 aa) was incorporated into ExoBp to ensure efficient SLC1A5 recognition while minimizing steric hindrance. Taken together, we build the dual-tag recombinant protein (His-Tag)-(Strept-Tag II)-RGD4C-G_4_S-hBD3 (23-67)-(GS)_3_-Syncytin-1 (21-150)-EA_3_K-CP05 with a predicted molecular weight of 28.6 kDa, for affinity to exosome surface proteins (Fig. 3A). The predicted structure of ExoBp suggests that each region adopts an independent spatial configuration without mutual shielding or steric interference (Fig. 3A).

### Expression and refolding challenges of ExoBp

The ExoBp construct was expressed in *E. coli* BL21(DE3); however, the protein accumulated predominantly in inclusion bodies, necessitating denaturation for solubilization and purification via nickel affinity chromatography (Fig. 3B to D). Refolding trials via dialysis into native buffers resulted in obvious aggregation. By refolding protein on Ni-NTA *in situ*, the eluted protein had high purity but low yield (Fig. 3E). These results suggest that the multidomain architecture of ExoBp, particularly the inclusion of peptides (RGD4C and CP05), and hBD3, an antimicrobial domain whose high accumulation is toxic to host [40], may hinder proper folding in bacterial systems. Further optimization of refolding conditions is required to recover functional protein.

### Immobilization of ExoBp on magnetic beads

**Fig. 4.**
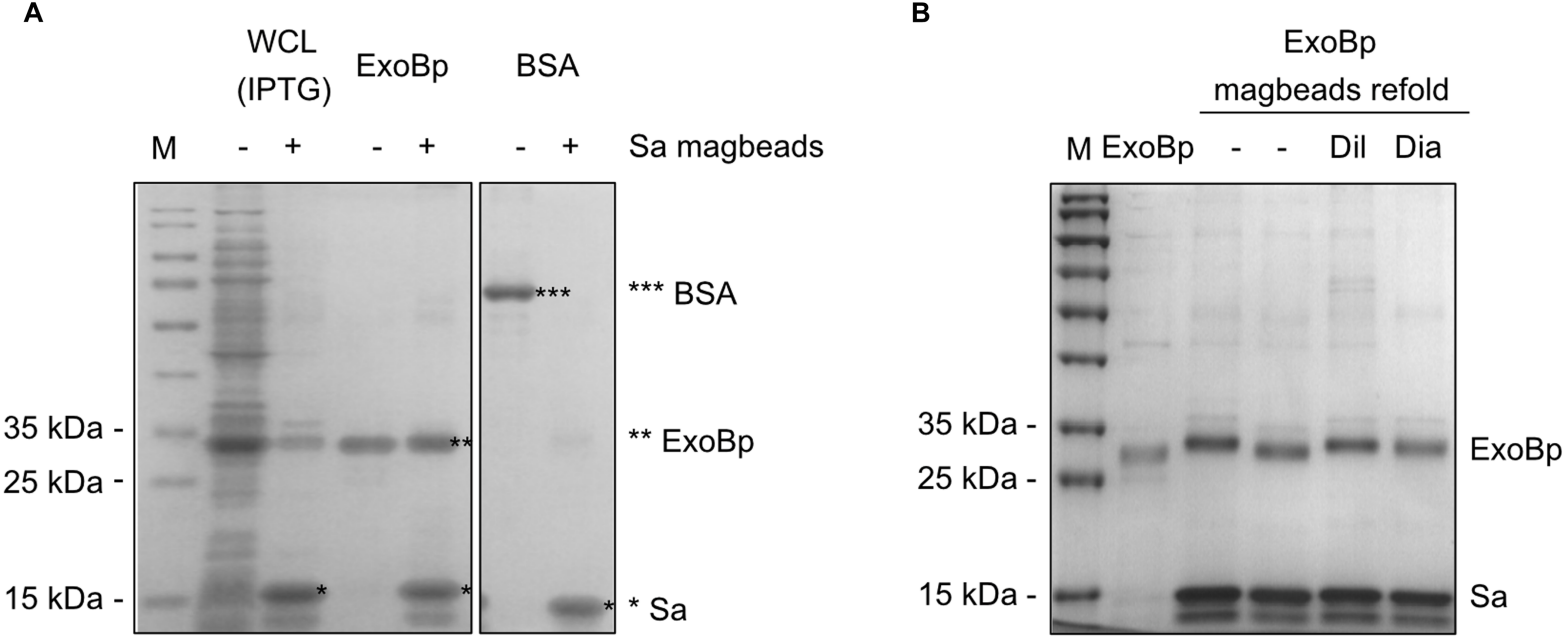
Immobilization ExoBp on magnetic beads. (A) Detection of immobilization of protein on streptavidin (Sa) magnetic beads. Whole cell lysis (WCL) after induction by IPTG or purified ExoBp incubated with Sa magnetic beads. After washing and collection, beads were analyzed by SDS-PAGE. Bovine serum albumin (BSA, 1 μg) was used as control. (B) Detection of refold ExoBp in site on magnetic beads. Dil, refold by dilution method; Dia, refold by dialysis method.

Despite refolding challenges, denatured ExoBp retained Strept-Tag II functionality, enabling its immobilization on streptavidin (Sa)-coated magnetic beads. Incubation of Sa beads with urea-solubilized ExoBp resulted in efficient binding (~20 μg/mg beads), as confirmed by SDS-PAGE (Fig. 4A). Control experiments with BSA showed minimal nonspecific adsorption. Notably, ExoBp from crude bacterial lysates also bound selectively to Sa beads, though with minor coimmobilization of host proteins (Fig. 4A). Post-immobilization refolding attempts via dialysis or rapid dilution into PBS preserved bead-bound ExoBp stability, confirming the feasibility of *in situ* refolding on the magnetic matrix with bare protein falling from beads (Fig. 4B). Thus, we have constructed magnetic beads immobilizing a recombinant protein containing 4 binding sites of exosome surface biomarkers.

### ExoBp beads affinity to exosome

**Fig. 5.**
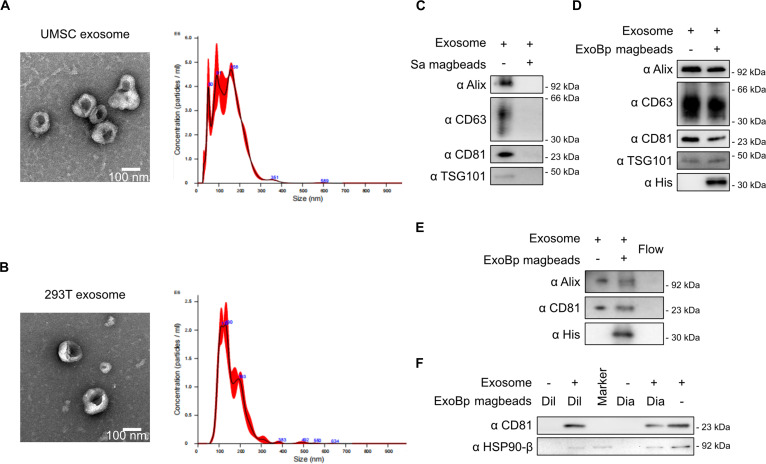
Enrichment of exosome by ExoBp beads. (A) Transmission electron microscopy (TEM) image and nanoparticle tracking analysis (NTA) of UMSC-derived exosome (UMSC exosome). (B) TEM image and NTA of 293T cell-derived exosome (293T exosome). (C) Exosome marker detection streptavidin (Sa) beads harvested after incubation of UMSC-derived exosome with Sa beads. (D) Exosome marker detection after enrichment of UMSC-derived exosome by ExoBp beads. (E) Exosome marker detection after enrichment of 293T-derived exosome by ExoBp beads. Flow, flow through after incubation of 293T-derived exosome and ExoBp beads. (F) Exosome marker detection after enrichment of UMSC-derived exosome by ExoBp beads that refolded in site by dilution or dialysis method. Dil, refold by dilution method; Dia, refold by dialysis method.

To validate exosome-binding capacity, refolded ExoBp beads were incubated with exosomes isolated from human umbilical cord-derived mesenchymal stem cells (hUMSCs) and 293T cells. The exosome exhibited a characteristic saucer-like morphology and a nano-scaled particle distribution (Fig. 5A and B). After incubation, beads were washed and analyzed for exosomal markers via Western blot. We validated no detectable nonspecific binding of exosome by Sa magbeads (Fig. 5C). ExoBp beads exhibited robust enrichment of UMSC- and 293T-derived exosomes (Fig. 5D and E). Binding capacity remained consistent across refolding methods (dialysis vs. dilution), with no difference in UMSC-derived exosome enrichment (Fig. 5F). These results demonstrate that ExoBp-functionalized magnetic beads efficiently capture exosomes, offering a scalable and versatile tool for exosome isolation.

## Discussion

Affinity enrichment of exosome presents advances in specificity, purity, and convenience. Exosome surface markers including CD9, CD63, CD81, and EpCAM were used as binding target for immune- or aptamer-affinity enrichment of exosome [33,41]. Other affinity strategies, lectin (concanavalin A) immobilized magnetic nanoparticle targeting reduced terminal α-d-mannose and α-d-glucose residues of glycolipids and glycoproteins on the surface of exosomes, or other exosome lipid targeting tools were also developed for enrichment of exosome [42,43]. However, because of the heterogeneity of exosome surface components, only a specific subset of exosome or EVs can be enriched, shielding the overall characteristic of exosome and contaminating by other EVs. The combination of multiple affinity tools against CD9, CD63, CD81, and EpCAM improves exosome capture, but the high cost of commercial antibodies relative to homemade recombinant protein limits its wide applications.

In this study, the ExoBp magnetic bead system leverages a multitarget approach to overcome the heterogeneity of exosome surface markers, enabling broad-spectrum exosome enrichment at less cost and with convenience. While challenges in recombinant protein refolding require further optimization, the platform’s ability to immobilize functional ExoBp and capture exosomes underscores its potential for diagnostic and therapeutic applications. However, this “proof of concept” study has own limits. This platform has not been used for exosome isolation from nature fluid or cell culture medium. Besides, we did not perform further mechanical or comparative studies, such as generating mutant domains to assess the mininal number of peptides sufficient for exosome capture, or comparing with commercial tools of exosome isolation. Moreover, it would be better for screening more efficiency recombinant proteins by constructing a pool of recombinant proteins containing different peptides that recognize exosome surface markers, or by using a direct evolution method.

## Data Availability

Data are provided within the manuscript or the Supplementary Materials.
