# Supplementary material for: Engineering Magnetic Beads for Affinity Enrichment of Exosomes
Source: Comput Struct Biotechnol J. 2026 Jul 22;35(1):0170. doi: 10.34133/csbj.0170 (PMC13389044; doi:10.34133/csbj.0170)

**Supplementary Information**

Engineering magnetic beads for affinity enrichment of exosome

Xu Wang ^1, 2, *^, Baiqing Li ^2^, Di Wu ^1^, Xiaotong Cen ^1^, Dajiang Qin ^1, 3, *^

1. Key Laboratory of Biological Targeting Diagnosis, Therapy and Rehabilitation of Guangdong Higher Education Institutes, The Fifth Affiliated Hospital, Guangzhou Medical University, Guangzhou, China.
2. Guangzhou National Laboratory, Guangzhou, China.
3. Centre for Regenerative Medicine and Health, Hong Kong Institute of Science & Innovation, Chinese Academy of Sciences, Hong Kong SAR, China.

*Corresponding author:Xu Wang: [wang_xu2@gzlab.ac.cn](mailto:wang_xu2@gzlab.ac.cn); Dajiang Qin: [qin_dajiang@gzhmu.edu.cn](mailto:qin_dajiang@gzhmu.edu.cn).

**Table of contents:**

Supplementary Tables

Tab. S1. General and universal biomarker membrane of exosome and their binding peptide or protein.

Tab. S2. Sequence information of ExoBp.

Raw files of gel and WB

**Tab. S1. General and universal biomarker membrane of exosome and their binding peptide or protein.**

| **Protein/membrane of exosome** | **Binding peptide/protein** | **Amino acid/domain** | **Ref** |
| --- | --- | --- | --- |
| CD63 | CP05 | CRHSQMTVTSRL | [1] |
| CD9 | CD9-BP | RSHRLRLH | [2] |
| CD81 | p_E2-S1 | INSTALNCNESLNTGWLAGLFYQ | [3] |
|  | C25 | CSPQYWTGPAC | [4] |
| CD47 | 4N1K | KRFYVVMWKK | [5] |
|  | pep-20 | AWSATWSNYWRH | [6] |
| SLC3A2 | hBD3 | GIINTLQKYYCRVRGGRCAVLSCLPKEEQIGKCSTRGR KCCRRKK | [7] |
| ITGB1 | RGD4C peptide | ACDCRGDCFCG | [8] |
| BSG (CD147) | AP-9 peptide | YKLPGHHHHYRP | [9] |
| SLC1A5 | Syncytin-1 | Surface domain | [10] |
| Membrane | BP | RPPGFSPFR | [11] |
|  | G58 | GAPDH 58-100 | [12] |

**Tab. S2. Sequence information of ExoBp regions.**

| **Regions of Tag-ExoBp** | **ExoBp amino acid sequence (with Tags)** |  |
| --- | --- | --- |
| His-Tag  Strept-Tag II  RGD4C  G_4_S linker  hBD3  (GS)3 liner  Syncytin-1 (21-150)  EA3K liner  CP05 | HHHHHHSSGLVPRGSHMASMTGGQQMGRGSWSHPQFEKGALEVLFQGPACDCRGDCFCGGGGGSGIINTLQKYYCRVRGGRCAVLSCLPKEEQIGKCSTRGRKCCRRKKGSGSGSAPPPCRCMTSSSPYQEFLWRMQRPGNIDAPSYRSLSKGTPTFTAHTHMPRNCYHSATLCMHANTHYWTGKMINPSCPGGLGVTVCWTYFTQTGMSDGGGVQDQAREKHVKEVISQLTRVHGTSSPYKGLDEAAAKCRHSQMTVTSRL |  |

**Reference**

[1] Gao X, Ran N, Dong X, Zuo B, Yang R, Zhou Q, et al. Anchor peptide captures, targets, and loads exosomes of diverse origins for diagnostics and therapy. Sci Transl Med 2018;10:eaat0195. https://doi.org/10.1126/scitranslmed.aat0195.

[2] Suwatthanarak T, Tanaka M, Miyamoto Y, Miyado K, Okochi M. Inhibition of cancer-cell migration by tetraspanin CD9-binding peptide. Chem Commun (Camb) 2021;57:4906–9. https://doi.org/10.1039/d1cc01295a.

[3] Chang C-C, Hsu H-J, Yen J-H, Lo S-Y, Liou J-W. A Sequence in the loop domain of hepatitis C virus E2 protein identified in silico as crucial for the selective binding to human CD81. PLoS ONE 2017;12:e0177383. https://doi.org/10.1371/journal.pone.0177383.

[4] Cao J, Zhao P, Miao XH, Zhao LJ, Xue LJ, Qi ZT. Phage display selection on whole cells yields a small peptide specific for HCV receptor human CD81. Cell Res 2003;13:473–9. https://doi.org/10.1038/sj.cr.7290190.

[5] Leclair P, Lim CJ. CD47-independent effects mediated by the TSP-derived 4N1K peptide. PLoS One 2014;9:e98358. https://doi.org/10.1371/journal.pone.0098358.

[6] Wang H, Sun Y, Zhou X, Chen C, Jiao L, Li W, et al. CD47/SIRPα blocking peptide identification and synergistic effect with irradiation for cancer immunotherapy. J Immunother Cancer 2020;8:e000905. https://doi.org/10.1136/jitc-2020-000905.

[7] Colavita I, Nigro E, Sarnataro D, Scudiero O, Granata V, Daniele A, et al. Membrane protein 4F2/CD98 is a cell surface receptor involved in the internalization and trafficking of human β-Defensin 3 in epithelial cells. Chem Biol 2015;22:217–28. https://doi.org/10.1016/j.chembiol.2014.11.020.

[8] Koivunen E, Wang B, Ruoslahti E. Phage libraries displaying cyclic peptides with different ring sizes: ligand specificities of the RGD-directed integrins. Biotechnology (N Y) 1995;13:265–70. https://doi.org/10.1038/nbt0395-265.

[9] Liu S, Jin R, Wang M, Li G. Nanoparticle Delivery of CD147 Antagonistic Peptide-9 Protects against Acute Ischemic Brain Injury and tPA-Induced Intracerebral Hemorrhage in Mice. ACS Appl Bio Mater 2020;3:1976–85. https://doi.org/10.1021/acsabm.9b01141.

[10] Štafl K, Trávníček M, Kučerová D, Pecnová Ľ, Krchlíková V, Gáliková E, et al. Heterologous avian system for quantitative analysis of Syncytin-1 interaction with ASCT2 receptor. Retrovirology 2021;18:15. https://doi.org/10.1186/s12977-021-00558-0.

[11] Gori A, Romanato A, Bergamaschi G, Strada A, Gagni P, Frigerio R, et al. Membrane‐binding peptides for extracellular vesicles on‐chip analysis. Journal of Extracellular Vesicles 2020;9:1751428. https://doi.org/10.1080/20013078.2020.1751428.

[12] Dar GH, Mendes CC, Kuan W-L, Speciale AA, Conceição M, Görgens A, et al. GAPDH controls extracellular vesicle biogenesis and enhances the therapeutic potential of EV mediated siRNA delivery to the brain. Nat Commun 2021;12:6666. https://doi.org/10.1038/s41467-021-27056-3.

**
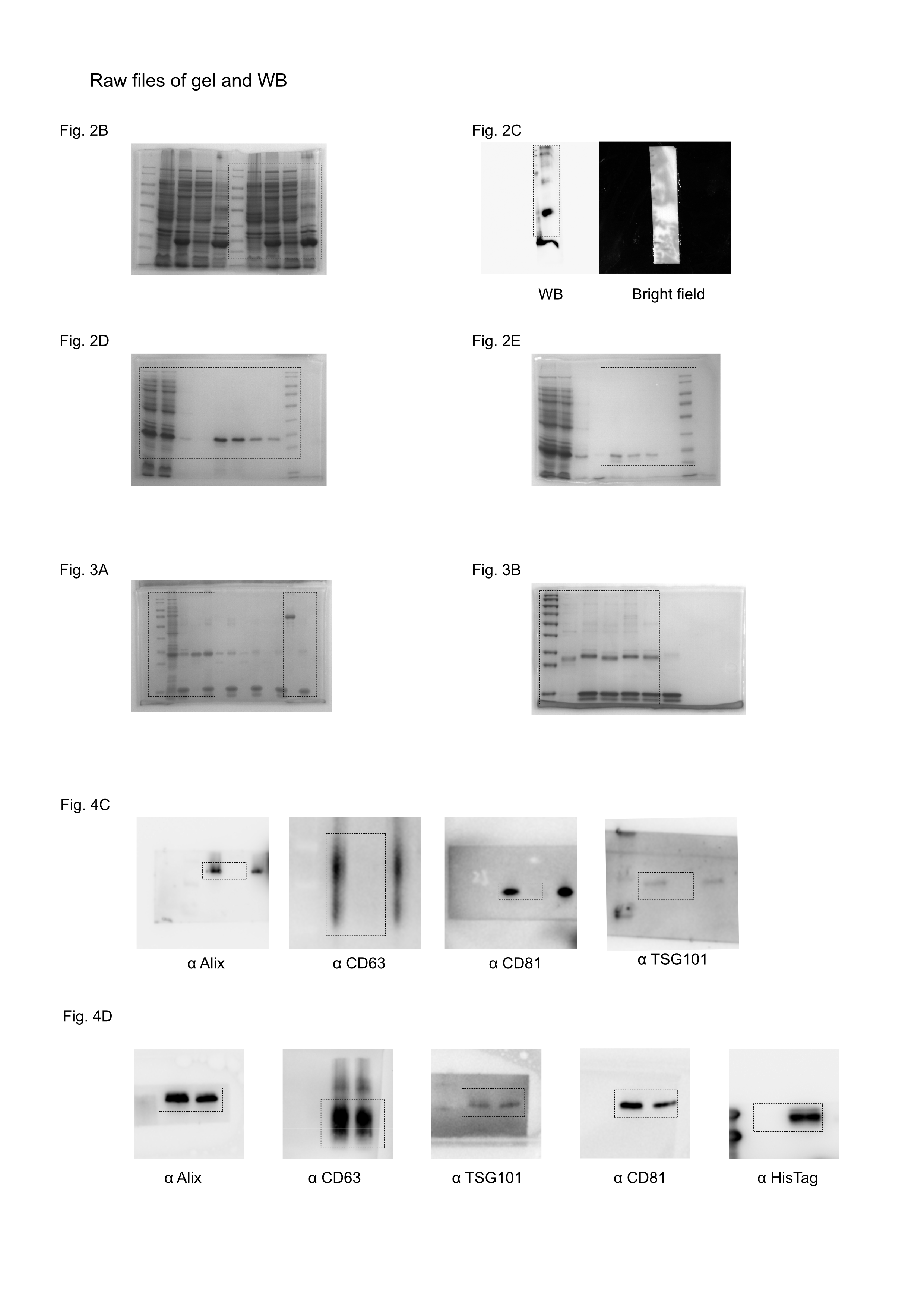
**


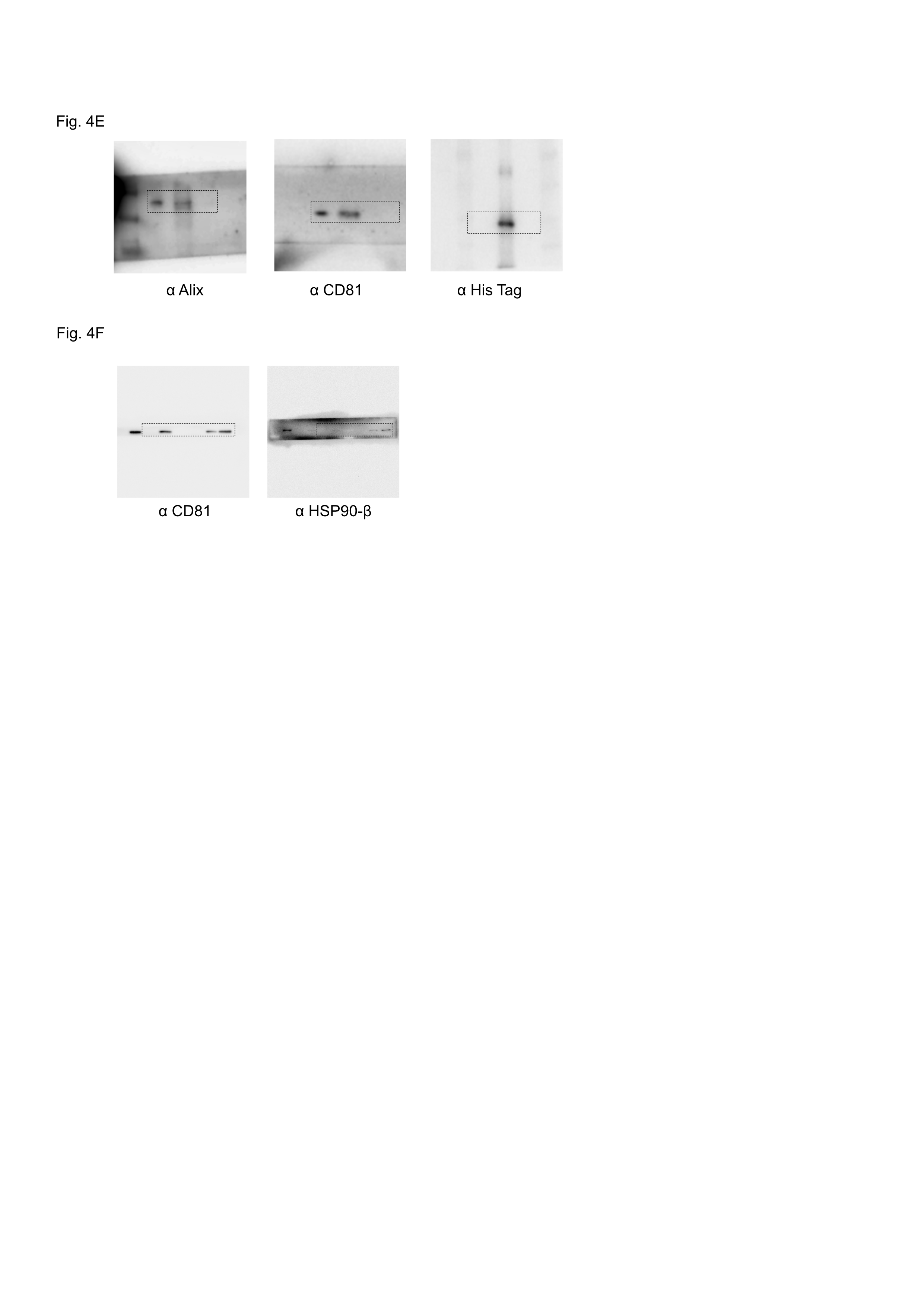

Supplement: Supplementary 1 — Tables S1 and S2 [file csbj.0170.f1.docx]
